# Welcome to *Source Code for Biology and Medicine*

**DOI:** 10.1186/1751-0473-1-1

**Published:** 2006-10-12

**Authors:** Leif E Peterson, Emmanuel C Ifeachor

**Affiliations:** 1 Department of Medicine; Department of Molecular & Human Genetics, Baylor College of Medicine, Houston, Texas, US; 2 School of Computing, University of Plymouth, Plymouth, UK

## Abstract

This editorial introduces *Source Code for Biology and Medicine*, a new journal for publication of programming source code used in biology and medicine. *Source Code for Biology and Medicine *is an open access independent journal published by BioMed Central. We describe the journal aims, scope, benefits of open access, article processing charges, competing interests, content and article format, peer review policy and publication, and introduce the Editorial Board.

## Aim and scope

*Source Code for Biology and Medicine *is a peer-reviewed open access, online journal that publishes articles on source code employed over a wide range of applications in biology and medicine. The aim of the journal is to publish source code for distribution and use in the public domain in order to advance biological and medical research. Through this dissemination, it may be possible to shorten the time required for solving certain computational problems for which there is limited source code availability or resources. Fundamentally, the overarching computation-related goals of the journal are to:

• Increase productivity among source code users working on problems of public and environmental health importance

• Reduce discovery times in molecular and genomic sciences

• Reduce search times for source code applied in biological and medical research

• Provide a historical reflection of source code applied in various fields

• Serve as a repository for source code

The scope of *Source Code for Biology and Medicine *includes workflow and source code for data integration, data fusion, gene regulatory networks, molecular pathway and drug discovery, protein structure, biological sequence analysis, signal processing, geospatial information systems, patient management systems, decision support systems, social network systems, environmental risk and dose modeling, supervised and unsupervised classification, text and media mining, and parametric/non-parametric numerical methods. Publications in *Source Code for Biology and Medicine *may involve the following areas:

• Biometrics

• Biostatistics

• Biopatterns

• Biophysics

• Bioinformatics

• Ecology

• Environmental health

• Health physics

• Medical physics

• Medical informatics

• Physics in medicine

• Psychometrics

• Toxicology

• Vital statistics

## Why an open access journal for *Source Code for Biology and Medicine *is needed

Computer algorithms have been the primary source of quantitative results in biology and medicine for decades. Unfortunately, publication of the specific codes used has never been a major enterprise, so there is not a large body of information on the implemented codes. Several journals on computers in biomedicine now exist; however, they are focused on algorithm design, results, and benchmarking, and not specifically on publication of the source code. An online open access journal which requires publication of the computer source codes used in biomedical applications meets a great and growing demand, since the journal would serve as a historical repository for code and also provide direct access to codes for future use. Direct access to source code can substantially accelerate scientific discovery in the biomedical disciplines.

## *Source Code for Biology and Medicine *is an open access journal

Under the open access publishing model, all articles become freely and universally accessible online, which means that access to articles in *Source Code for Biology and Medicine *does not require an annual subscription fee or online purchase of articles. Open access also guarantees that authors hold the copyright for their work and grant anyone the right to reproduce and disseminate the article. Open access articles are also archived in PubMed Central [1] as well as repositories at the University of Potsdam [2] in Germany, INIST [3] in France, and e-Depot [4], the National Library of the Netherlands' digital archive of all electronic publications.

There are many benefits of open access publishing. These include the widest dissemination of published work with few barriers, which casts a wider net than print and online subscription-based journals. Recently, Eysenbach [5] studied open access vs. non-open access articles published by *PNAS *from June 8, 2004 to December 20, 2004. Using a logistic regression model, and controlling for potential confounders, the odds ratio for citation of open access *PNAS* articles 4–10 months after publication compared with non-Open-Access *PNAS* articles was 2.1 (95% CI, 1.5–2.9). The odds ratio increased to 2.9 (95% CI, 1.5–5.5) for 10–16 months after publication. Invariably, open access results in more downloads and citations, and can therefore increase Impact Factor [6,7]. Online free access also enhances literature searching, since direct access to articles is not constrained by an institutional library's budget. Open access articles are accessible to all taxpayers, and not just those with a subscription or those who purchase online. The US National Institutes of Health (NIH) and the Wellcome Trust in the UK currently have policies for grant awardees to release their research to the public as soon as possible after publication [8-10]. Lastly, scientists in countries with harsh economic environments for which subscription and online purchase is impossible can access articles directly.

## Article processing charges

An article processing charge (APC) is required for each article accepted for publication in *Source Code for Biology and Medicine*. APCs defray costs for world-wide barrier-free access to peer-reviewed papers, developing and maintaining electronic tools for peer review and publication, preparation of papers in various formats for on-line publication, securing citation inclusion in PubMed, inclusion in PubMed Central archives, inclusion into CrossRef for citations in other electronic journals. APCs also ensure transparency and allow publishers to compete to provide the best service at the best price. By coupling the cost of publication to research budgets, APCs ensure that the journal publishing system can scale to cope with an ever-increasing volume of research.

Many institutions [11] and research funders [12] will cover APCs for their researchers. In addition, in exceptional circumstances APCs can be waived for authors who do not belong to a BioMed Central member institute and who cannot cover the publishing costs themselves. It warrants noting that APCs are required in order to sustain open access.

## Competing interests

The Editors-in-Chief and the majority of Editorial Board members of *Source Code for Biology and Medicine *are academic researchers at non-profit universities mostly supported by Government research grants. As such, there is no conflict of interest during the review process or in the publication of articles appearing in *Source Code for Biology and Medicine*. Overall, the Editors-In-Chief and Editorial Board members are genuinely interested in publishing high-quality articles on computation in the biological and medical fields for the non-profit reader community.

## Content of *Source Code for Biology and Medicine*

The types of articles published in *Source Code for Biology and Medicine *are as follows:

• **Research article: **describes novel implementation of source code used for acquiring new results not previously published. Requires benchmarking with other algorithms and submission of complete source code files with compiled executables, libraries, or class modules (object-oriented programming is the preferred programming style).

• **Methodology article: **describe first-time publication of source code for a common application for which code is not widely available in peer-reviewed biomedical literature. Requires submission of complete source code files with compiled executables, libraries, or class modules.

• **Brief reports: **Provide a synopsis of an applied algorithm without publication of the source code.

• **Software reviews: **covers the application of a published workflow or algorithm.

Manuscripts for Research or Methodology articles submitted to *Source Code for Biology and Medicine *should be formatted using the following section headers:

• Title page

• Abstract (350-word limit)

• Introduction/Background

• Methods

• Results and Discussion

• Conclusions

• System and Programming Language Requirements

• Copyright, Trademarks, Licensing, and Registration Requirements

• Availability

• Competing interests

• Authors' contributions

• Acknowledgements

• References

• Figure legends (if any)

• Tables and captions (if any)

• Description of additional data files

○ Source Code Listing

○ Bundled Data Description (if any)

○ Examples Runs (if any)

These sections may contain subsections. For example:

Methods

○ Particle energy binning (histograms)

○ Computation of factorial moments

## Peer review policy and publication

*Source Code for Biology and Medicine *aims at a turnaround time for manuscript reviews of two weeks. All manuscripts will be reviewed anonymously by at least two referees. Review criteria include scope and originality; linguistic quality; comprehensiveness in background history; review of previous research with references, completeness in descriptions of data and samples used and methods implemented; discussion of results in connection with data used and results from other studies; benchmarking with other algorithms using simulated and/or empirical data sets; comparison with other codes; referencing and citations; and adequacy of tables and figures. A description of availability is required. Acceptance/rejection criteria are: accept without change, accept with minor modifications, review after modifications, or reject (unsuitable for publication).

Once an article is accepted for publication, it will be published online immediately in *Source Code for Biology and Medicine*, and will be indexed shortly thereafter in PubMed and PubMed Central.

## Editorial board

The membership of the Editorial Board for *Source Code for Biology and Medicine *can be viewed online [13].
